# Inter-tissue communication of mitochondrial stress and metabolic health

**DOI:** 10.1093/lifemeta/load001

**Published:** 2023-01-07

**Authors:** Hanlin Zhang, Xinyu Li, Wudi Fan, Sentibel Pandovski, Ye Tian, Andrew Dillin

**Affiliations:** Department of Molecular and Cell Biology, Howard Hughes Medical Institute, University of California, Berkeley, Berkeley, CA 94720, USA; State Key Laboratory of Molecular Developmental Biology, Institute of Genetics and Developmental Biology, Chinese Academy of Sciences, Beijing 100101, China; University of Chinese Academy of Sciences, Beijing 100093, China; Department of Molecular and Cell Biology, Howard Hughes Medical Institute, University of California, Berkeley, Berkeley, CA 94720, USA; Department of Molecular and Cell Biology, Howard Hughes Medical Institute, University of California, Berkeley, Berkeley, CA 94720, USA; State Key Laboratory of Molecular Developmental Biology, Institute of Genetics and Developmental Biology, Chinese Academy of Sciences, Beijing 100101, China; University of Chinese Academy of Sciences, Beijing 100093, China; Center for Excellence in Animal Evolution and Genetics, Chinese Academy of Sciences, Kunming, Yunnan 650223, China; Department of Molecular and Cell Biology, Howard Hughes Medical Institute, University of California, Berkeley, Berkeley, CA 94720, USA

**Keywords:** mitochondria, inter-tissue communication, mitokine, metabolic health, quorum sensing

## Abstract

Mitochondria function as a hub of the cellular metabolic network. Mitochondrial stress is closely associated with aging and a variety of diseases, including neurodegeneration and cancer. Cells autonomously elicit specific stress responses to cope with mitochondrial stress to maintain mitochondrial homeostasis. Interestingly, mitochondrial stress responses may also be induced in a non-autonomous manner in cells or tissues that are not directly experiencing such stress. Such non-autonomous mitochondrial stress responses are mediated by secreted molecules called mitokines. Due to their significant translational potential in improving human metabolic health, there has been a surge in mitokine-focused research. In this review, we summarize the findings regarding inter-tissue communication of mitochondrial stress in animal models. In addition, we discuss the possibility of mitokine-mediated intercellular mitochondrial communication originating from bacterial quorum sensing.

## Introduction

Mitochondria are double-membrane-bound cellular organelles that are essential for a variety of biological processes in eukaryotic cells. Within this specialized structure, a proton gradient across the inner membrane is generated by the electron transport chain for ATP and/or heat production. Reactive oxygen species (ROS) are generated as by-products of electron transport and function as key signaling molecules. The mitochondrial matrix is the site of calcium storage and the biogenesis of fatty acids, amino acids, nucleotides, and heme. These basic biochemical functions and components endow mitochondria a fundamental role in regulating basic cellular processes, including cell growth, innate immune responses, cell differentiation, and cell death.

Due to mitochondria’s metabolically active nature and its role in facilitating essential cellular functions, mitochondrial homeostasis is maintained in cells via multiple quality control mechanisms. The mitochondrial unfolded protein responses (UPR^MT^s) are transcriptional responses activated during mitochondrial stress to up-regulate the expression of certain chaperones, proteases, and detoxifying enzymes to help the stressed mitochondria to recover [1]. Specific UPR^MT^ mechanisms have been most thoroughly identified in *Caenorhabditis elegans*. Currently, the most widely accepted working model of UPR^MT^ activation is mediated by activating transcription factor associated with stress-1 (ATFS-1), which is a key transcription factor that activates UPR^MT^ in worms. It contains a nuclear localization sequence (NLS) and a mitochondria-targeting sequence (MTS), and thus the mitochondria and the nucleus compete for this protein [2]. Under homeostatic conditions, the MTS prevails, and thus ATFS-1 is preferentially imported into mitochondria, where it is degraded by the protease long-filament phenotype (LON) in the mitochondrial matrix [2]. Mitochondrial stress is often associated with reduced mitochondria import efficiency, leading to reduced import of ATFS-1 into the mitochondria and thus nuclear enrichment of ATFS-1 followed by activation of the UPR^MT^ transcriptional program [2].

In mammals, the transcription factor ATF5 was identified as a homolog of worm ATFS-1 [3]. ATF5 also contains an NLS and a putative MTS, and functions in a similar way to ATFS-1 to promote the transcription of UPR^MT^ genes [3]. Importantly, mammalian ATF5 is sufficient to rescue the UPR^MT^ deficiency in *atfs-1* mutant worms, indicating the functional similarity between ATF5 and ATFS-1 [3]. Moreover, mammalian cells have adopted the integrated stress response (ISR) as an alternative way to relieve mitochondrial stress. Mitochondrial stress in mammalian cells leads to activation of the mitochondrial protease Overlapping Activity With M‑AAA Protease (OMA1), which cleaves the mitochondrial protein DAP3 binding cell death enhancer‑1 (DELE1) to promote its translocation from the mitochondria to the cytosol [4, 5]. Cytosolic DELE1 binds to and activates the kinase heme-regulated inhibitor (HRI), which is one of the four kinases that are known to phosphorylate eukaryotic translation initiation factor 2α (eIF2α). Phosphorylation of eIF2α leads to suppression of eIF2α’s function and inhibition of canonical translation initiation. However, such inhibition can paradoxically promote the translation of specific mRNAs due to their special upstream open reading frames [6]. Under optimal conditions of translation, these short regulatory upstream open reading frames can be efficiently translated, leading to suppression of translation of the downstream gene-coding open reading frame. One of those genes whose translation is activated by phospho-eIF2α is activating transcription factor 4 (ATF4), which is a key transcription factor that promotes the expression of stress response genes, including mitochondrial chaperones. The ISR pathway can sense stress from multiple cellular processes via kinases including double stranded RNA-dependent protein kinase (PKR)-like ER kinase (PERK) (ER stress), PKR (viral stress), general control nonderepressible 2 (GCN2) (amino acids starvation), and HRI (low heme, and other types of stress such as mitochondrial stress) [6]. Notably, in mammalian cells, there is a convergence of different stress signals to the same eIF2α-ATF4 ISR pathway. This may not be too surprising considering that the ISR leads to very basic adaptations, such as improved mitochondrial homeostasis, that are always fundamental to maintaining cellular functions under various stress conditions.

Interestingly, mitochondrial stress responses can also occur in cells that do not directly experience mitochondrial stress. In such cases, a subset of cells with induced mitochondrial stress responses can secrete molecules to induce corresponding stress responses or metabolic adaptations in recipient cells. This phenomenon was first reported in *C. elegans* [7], and subsequently widely observed in mammals. Various forms of cellular autonomous mitochondrial stress responses have been thoroughly summarized by recently published papers [1, 8, 9]. In this review, we will discuss the non-autonomous perspective of mitochondrial stress responses, focusing on the secreted signaling molecules that mediate these responses, namely, the mitokines.

## Inter-tissue communication of mitochondrial stress in *C. elegans*

**Figure 1:**
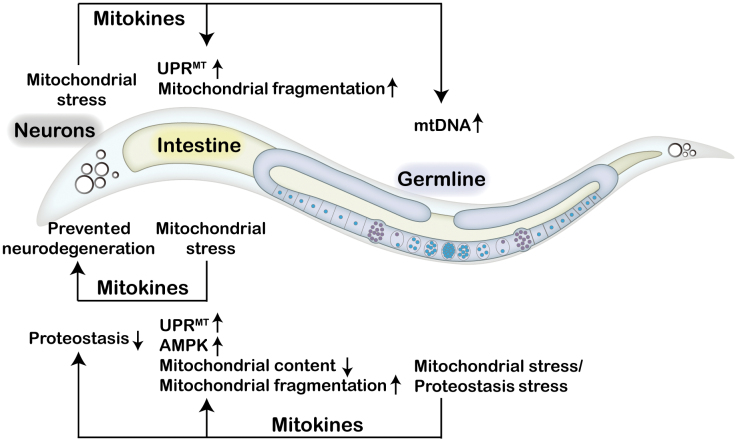
Mitokines coordinate inter-tissue communication of mitochondrial stress in *Caenorhabditis elegans.* In *C. elegans*, neurons under mitochondrial stress secrete mitokines to activate the UPR^MT^ and promote mitochondrial fragmentation in the intestine and to elevate mtDNA levels in the germline. Intestinal cells experiencing mitochondrial stress protect animals from neurodegeneration via the action of unknown mitokines. Mitochondrial stress or proteostasis failure in the germline leads to the release of mitokines that trigger the UPR^MT^ and AMPK signaling, reducing mitochondrial content and promoting mitochondrial fragmentation in the intestine, while also promoting protein aggregation in the intestine and neurons.

Severe mitochondrial damage might be detrimental; however, mild mitochondrial stress has the potential to improve longevity through the activation of protective mitochondrial stress responses in the nucleus, as revealed by various studies spanning different species [10–14]. Mitokine-mediated communication of mitochondrial stress between tissues may prepare the organism to cope with the everchanging environment. Extensive research on mitokines has been performed in *C. elegans* since the first report of the cell non-autonomous nature of electron transport chain-mediated longevity [7]. Mitochondrial perturbation in neurons or in the germline can be perceived by the intestine, leading to the induction of the UPR^MT^ in intestinal cells [7, 15] (Fig. 1). Since the nervous system does not directly innervate the intestine in *C. elegans*, it is proposed that neuronal mitochondria promote the release of secreted mitokine signals in response to proteotoxic stress [7]. To date, multiple mitokines have been identified in *C. elegans*, including neurotransmitters, neuropeptides, insulin-like peptides, and Wnt/EGL-20 [16–19].

### Serotonin

Polyglutamine (polyQ) tract repeats are a sequence of multiple consecutive glutamines. PolyQ-containing proteins tend to aggregate and are associated with neurodegenerative diseases, such as Huntington’s disease. In *C. elegans*, neuron-expressed polyQ40 associates with mitochondria and affects mitochondrial function and fitness. Intriguingly, neuronal expression of polyQ40 is sufficient to elicit a protective form of the UPR^MT^ in distal tissues. Calcium-dependent dense core vesicles (DCVs) release is required for mitokine signaling in the neuronal polyQ40 mitochondrial stress model [16]. A further candidate screen identified serotonin, a cargo of DCVs, as a required factor for cell non-autonomous mitochondrial stress signaling [16]. Serotonin is a monoamine neurotransmitter synthesized by the tryptophan hydroxylase (TPH)-mediated reaction. In mammals, serotonin regulates multiple functions, including mood, reward, learning, memory, and sleep. In *C. elegans*, serotonin controls food-related behavior and fat regulation [20]. Although TPH-1-regulated serotonin synthesis is necessary for the induction of the inter-tissue UPR^MT^ and the application of exogenous serotonin is sufficient to rescue cell non-autonomous UPR^MT^ signaling in neurosecretion-defective mutant worms, supplementation of serotonin is not sufficient to activate the UPR^MT^ in the intestine [16]. These results indicate that alternative signaling molecules are required for cell non-autonomous UPR^MT^ signaling. Moreover, the receptor of serotonin and the downstream signaling pathway required for cross-tissue mitochondrial stress communication remain to be determined.

### FMRFamide-like peptide (FLP)-2

The neuropeptide FLP-2 also serves as a mitokine to control inter-tissue UPR^MT^ activation. FLP-2 regulates locomotion arousal in *C. elegans* [21]. Using a different neuronal mitochondrial stress model, Shao *et al*. reported that neuronal-specific knockout of the mitochondrial protease gene *spg-7* induces cell non-autonomous UPR^MT^ [18]. A neuron sub-circuit composed of three sensory neurons, chemosensory neuron ASK, amphid sensory neuron (AWA neuron), and thermosensory neuron AWC, and one interneuron, Amphid interneuron (AIA), was identified to be required for perceiving and transducing neuronal mitochondrial stress in this model [18]. The neuropeptide FLP-2 functions in the AIA interneuron to mediate the cell non-autonomous UPR^MT^ upon neuronal knockout of *spg-7,* and neuron-specific FLP-2 overexpression is sufficient to activate an inter-tissue mitochondrial stress response [18]. However, neuronal overexpression of FLP-2 cannot promote longevity despite the induced UPR^MT^ in the periphery, indicating that the polyQ40 model and the *spg-7* knockout model may induce distinct signaling events apart from the shared UPR^MT^ induction phenotype [18]. Notably, serotonin is also required for the induction of peripheral UPR^MT^ in the neuronal *spg-7* mutant model [16]. The relationship between FLP-2 and serotonin in the regulation of the cell non-autonomous UPR^MT^ remains to be determined.

### Wnt

The Wnt protein is a well-studied morphogen controlling multiple developmental processes, including cell fate decision, cell migration, body axis formation, and organogenesis, across species [22]. Interestingly, Wnt has also been found to be required for the inter-tissue communication of mitochondrial stress in worms. A genetic screen utilizing the neuronal polyQ40 model found that retromer-dependent Wnt signaling is required for the induction of cell non-autonomous UPR^MT^ [17]. The retromer complex is a highly conserved multi-subunit complex mediating the retrograde transport of cargos between endosomes and the trans-Golgi network [23]. Various cargos, including Wnt binding protein Wntless (WLS)/MIG-14, are retrieved by the retromer complex. WLS/MIG-14 is the Wnt secretion factor responsible for the Wnt ligand EGL-20 secretion and long-range signaling [24]. Genetic mutations of the retromer complex or WLS/MIG-14 prevent Wnt secretion and thereby suppress cell non-autonomous UPR^MT^. There are five Wnt ligands in *C. elegans*, and EGL-20 is the only Wnt ligand specifically required for cell non-autonomous UPR^MT^ signaling. Wnt/EGL-20 is expressed in a group of posterior cells in *C. elegans*, including a subset of neurons. During early developmental stages, Wnt/EGL-20 needs to be secreted and thus forms a posterior-to-anterior concentration gradient to control several developmental events, such as neuron migration.

During Wnt’s folding in the endoplasmic reticulum (ER), its conserved cysteines form disulfide bonds, which are critical for the structure and the activity of the Wnt protein. The protein disulfide isomerase PDI-6 mediates Wnt/EGL-20 secretion, which is required for the inter-tissue communication of the UPR^MT^ [25]. While there are no significant changes in the mRNA level of Wnt/EGL-20 under neuronal mitochondrial stress, the mRNA and protein levels of PDI-6 are significantly increased [17, 25]. Upregulated PDI-6 potentially elevates the level of functional Wnt/EGL-20 to propagate a systemic mitochondrial stress response. The canonical Wnt signaling pathway involves activation of the transcriptional co-activator β-catenin/BAR-1 and the transcription factor T-cell factor (TCF)/POP-1 in the receiving cells, which are necessary for the induction of inter-tissue UPR^MT^. Notably, the neuronal overexpression of Wnt/EGL-20 or PDI-6 is sufficient to activate the cell non-autonomous UPR^MT^ in the intestine and extend lifespan in *C. elegans* [17, 25]. Furthermore, neuronal Wnt/EGL-20-induced cell non-autonomous UPR^MT^ requires the neurotransmitter serotonin [17], indicating that neurosecretion coordinates Wnt/EGL-20 signaling to regulate mitochondrial stress communication. Interestingly, the overexpression of Wnt/EGL-20 solely in serotonergic neurons is also sufficient to induce a peripheral UPR^MT^ [17]. While it is likely that serotonin and Wnt/EGL-20 act in parallel to regulate the systemic mitochondrial stress response, it remains to be investigated how Wnt/EGL-20 and serotonin are coordinated to control organismal UPR^MT^.

### Other neurotransmitters and neuropeptides

Specific neurons play an essential role in coordinating the systemic UPR^MT^ in *C. elegans*. A pair of chemosensory neurons, ADL neurons, is required for the propagation of mitokine signals. Notably, the G-protein coupled receptor (GPCR) SRZ75 and the downstream Gαq signaling is critical for the ADL neurons to mediate the cell-non-autonomous UPR^MT^. Activating Gαq signaling in ADL neurons upregulates genes involved in neuropeptides processing, thus potentially promoting the release of neuropeptides. ADL-specific knockdown of neuropeptide genes, such as *neuropeptide-like protein (nlp)-55*, *nlp-67*, *nlp-76*, *dauer formation (daf)-28,* and *ins-14*, suppresses the induction of peripheral UPR^MT^, and ADL-specific overexpression of *ins-14* and *nlp-76* is sufficient to activate UPR^MT^ in the intestine. Furthermore, activation of Gαq signaling in ADL neurons improves resistance to pathogen infection, promotes proteostasis in the muscle, alters the mitochondrial morphology in the muscle and hypodermis, reduces oxygen consumption rates, and decreases fat storage in the intestine [26]. While the improved pathogen resistance and muscular proteostasis are mediated by neuropeptides, it remains to be determined whether neuropeptides are involved in the altered lipid metabolism and mitochondrial dynamics.

Mitochondria are highly dynamic organelles. The balance between mitochondrial fission and fusion is essential for mitochondrial homeostasis. Recently, it was reported that neuronal mitochondrial dynamics control the mitochondrial stress response and mitophagy non-autonomously. Loss of neuronal FZO-1, the sole *C. elegans* mitofusin, induces a non-autonomous UPR^MT^ that requires multiple neurotransmitters and neuropeptides, including acetylcholine, tyramine, glutamate, serotonin, and two insulin-like peptides, INS-27 and INS-35 [19]. Exogenous serotonin restores cell non-autonomous UPR^MT^ in glutamate- and tyramine-deficient mutants, but not in acetylcholine-deficient mutants, indicating that serotonin might act downstream or in parallel to glutamate and tyramine signaling, but upstream of cholinergic signaling, in the cell non-autonomous UPR^MT^ signaling pathway [19]. It should be noted that one of these signal molecules, tyramine, is not required for intestinal UPR^MT^ induction in the neuronal polyQ40 model [16]. It is interesting to speculate that various neuronal mitochondrial stresses might be perceived and propagated via different neuron-secreted molecules.

Different forms of neuronal mitochondrial stress seem to employ distinct mechanisms to elicit the cell non-autonomous UPR^MT^. Besides genetic perturbations of neuronal mitochondria, the pharmacological inhibition of mitochondrial function through paraquat treatment can activate the UPR^MT^ through cell-autonomous and non-autonomous manners. The GPCR follicle stimulating hormone receptor (FSHR)-1 functions in neurons to regulate the peripheral UPR^MT^ in response to paraquat treatment [27]. Mitochondrial ceramide produced by SPTL-1/serine palmitoyltransferase and sphingosine-1-phosphate (S1P) produced by SPHK-1/sphingosine kinase are critical for the activation of the UPR^MT^ in the intestine [28, 29]. Upon mitochondrial stress, SPHK-1 is targeted to mitochondria. Yet, the mechanism by which subsequent S1P production triggers the UPR^MT^ remains unknown. Furthermore, neuroendocrine signaling can regulate intestinal mitochondrial recruitment of SPHK-1 for the activation of UPR^MT^ [29]. UNC-31-mediated neuropeptide signaling, but not serotonin signaling, regulates intestinal SPHK-1 to control inter-­tissue UPR^MT^ activation, potentially through activating the GPCR FSHR-1 in the nervous system [27]. However, the identity of the neuropeptide and the downstream signaling pathway involving FSHR-1 that controls the cell non-autonomous UPR^MT^ remains elusive. Taken together, the nervous system plays an essential role in the regulation of the cell non-autonomous UPR^MT^. Further study on neuron-produced mitokine transmission and the downstream signaling mechanisms may shed new light on the systemic coordination of the mitochondrial stress response.

### Mitokine-induced metabolic adaptation in worms

The activation of the UPR^MT^ not only maintains mitochondrial proteostasis, but also regulates cellular metabolism [30]. Data derived from the chromatin immunoprecipitation, coupled with the DNA-sequencing (ChIP-seq), of the UPR^MT^ core transcription factor ATFS-1 suggest that the UPR^MT^ includes both proteostasis and metabolism genes. The metabolic pathways regulated by UPR^MT^ include glycolysis/gluconeogenesis, ribosome, oxidative phosphorylation (OXPHOS), the tricarboxylic acid cycle (TCA cycle), and autophagy. During the UPR^MT^, ATFS-1 represses the accumulation of nuclear-encoded OXPHOS and TCA cycle transcripts [31]. Furthermore, inactivation of some TCA cycle enzymes (e.g. aconitase dehydrogenase ACO-2 or isocitrate dehydrogenase IDHA-1) leads to citrate accumulation, which triggers the UPR^MT^ and promotes lipid accumulation. The UPR^MT^ transcription factor DVE-1 promotes the expression of the nuclear hormone receptor nuclear hormone receptor NHR-80. As a result, NHR-80 increases lipogenesis and lipid accumulation [32]. Notably, neuronal polyQ40-related stress impairs the oxygen consumption rate of the whole animal, indicating mitochondrial respiration dysfunction [16]. Intriguingly, perturbations in neuronal mitochondria also trigger alterations in peripheral mitochondrial morphology. Neuronal *fzo-1* RNAi induces peripheral mitochondrial fragmentation. Insulin signaling acts within the nervous system to control systemic mitochondrial morphology. Two insulin-like peptides, INS-27 and INS-35, act on DAF-2 (the sole insulin-like growth factor 1 receptor in *C. elegans*) to control the systemic UPR^MT^ and mitochondrial morphology. As a result, systemic UPR^MT^ activation and mitochondrial fragmentation confer resistance to pathogenic *Pseudomonas* infection [19]. Although cell-autonomous UPR^MT^ activation is associated with lipid accumulation and the mitokine serotonin controls fat in *C. elegans* [20, 32], whether mitokines regulate organismal lipid metabolism under mitochondrial stress remains to be determined.

### Other forms of inter-tissue communication of mitochondrial stress

Apart from receiving mitokines, intestinal cells also sense mitochondrial stress and release signals to the nervous system. For instance, rotenone exposure can damage mitochondrial complex I and thus triggers the immune pathway in the intestine. Intriguingly, activation of the immune pathway in the intestine protects rotenone-induced dopamine neuron degeneration [33]. However, the signaling mechanisms that mediate the intestine-to-neuron communication of mitochondrial stress remain unknown.

The germline is also involved in the coordination of the systemic mitochondrial stress response. Neuronal mitochondrial stress signals can be transmitted via the mitokine Wnt/EGL-20 signaling to increase mitochondrial DNA (mtDNA) levels in the germline, which causes proteostatic stress within mitochondria, and induce systemic mitochondrial stress response across multiple generations [34, 35]. Notably, loss of germline proteostasis not only impinges on the mitochondrial network of the germline, but also reduces the mitochondrial content of somatic tissues and subsequently activates the somatic UPR^MT^ through long-range Wnt/EGL-20 signaling. Germline proteostasis collapse promotes somatic mitochondrial fragmentation and protein aggregation in the soma, including neurons, intestine, and muscle [36]. Furthermore, germline-specific knockdown of the cytochrome c gene *cyc-2.1* activates the cell non-autonomous UPR^MT^ and AMP-activated protein kinase (AMPK) via unknown signals [15]. Further study is needed to elucidate the communication between somatic tissues and the germline for the systemic regulation of mitochondrial stress response.

## Mammalian mitokines and their roles in regulating organismal metabolism

After initial investigations in *C. elegans* of the nature of the mitokines released upon mitochondrial stress, mitokines in mammals have been extensively investigated and identified. In mammals, mitokine responses could serve as markers of disease severity and progression. Thus far, multiple mammalian mitokines have been identified, including fibroblast growth factor 21 (FGF21), growth differentiation factor 15 (GDF15), various mitochondrial-derived peptides (MDPs), adrenomedullin 2 (ADM2), and angiopoietin-like 6 (ANGPTL6) (Table 1).

**Table 1:** Mitokines in mammals

Mitokine name	Stress/expression inducers	Effects on cellular pathways and metabolism	Effects on aging
FGF21	Generally, acts as an anti-inflammatory factor in metabolism-related diseases Increased expression due to mitochondrial dysfunction	Sends signals non-autonomously to distal cells to reduce energy metabolism for non-essential functions Increases insulin sensitivity and thermogenesis in response to hepatic mitochondrial dysfunction Increases glucose catabolism in liver tissue	With the expression of aging-related genes, has the ability to delay cellular aging
GDF15	Increased expression to help prevent diet-induced hepatic steatosis and to regulate body fat Secreted to combat the inflammation associated with obesity-associated lysosomal stress	Suppresses organismal food and energy intake Increases the expression of certain hepatic enzymes to increase glucose catabolism and fatty acid metabolism. Decreases gene sets responsible for steroid hormone biosynthesis and lipid metabolism (allocating cellular energy towards more necessary mechanisms).	One of the most upregulated proteins in response to age-related disease and aging Serves protective roles for many tissues in response to stress and aging
MOTS-c	Induced by exercise in humans In mice, MOTS-c levels decrease after cold treatment *In vitro*, MOTS-c levels increase after glucose restriction, serum deprivation, and oxidative stress	Activates AMPK to improve energy metabolism and prevents DIO and insulin resistance Reduces visceral fat accumulation and hepatic steatosis in high-fat diet-fed mice Inhibits genes related to lipogenesis and increases glucose uptake, glucose utilization, fatty acid oxidation, and oxidative respiration Suppresses inflammation	Circulating levels decrease with age The m.1382A>C polymorphism of MOTS-c is associated with longevity in the Japanese population Protects against aging-related diseases by increasing senescence-associated secretory phenotypes
Humanin (HN)	In patients with Alzheimer’s disease, the serum level of HN is decreased. Reduced by insulin growth factor 1 and growth hormone	Has anti-apoptosis, anti-oxidation, anti-inflammatory, cytoprotective, and neuroprotective effects Protects against inflammation-related diseases, such as Alzheimer’s disease, type 2 d Diabetes, cardiovascular disease, and atherosclerosis	Conflicting literature on whether the level of HN increases [37, 38] or decreases [39, 40] with age Treatment of HN protects against age-related diseases Children of centenarians have higher HN than age-matched controls
Small Humanin-Like Peptides (SHLPs)	The inducer of SHLPs has yet to be investigated	Reduces apoptosis, decreases generation of ROS, improves mitochondrial metabolism, increases glucose uptake, suppresses hepatic glucose production, regulates leptin levels, and increases cell viability	May preserve mitochondrial function during aging and induces exercise-like adaptations Circulating levels decrease with age Protects against age-related diseases, such as Alzheimer’s disease
ADM2	Induced by mitochondrial stress and ATF4	Inhibits obesity-induced insulin resistance in mice	ADM levels are elevated in aged human brain ADM levels are decreased as mice age ADM knockout mice show better memory preservation during aging [41]
ANGPTL6	Induced by *Crif1* knockout Induced by OXPHOS dysfunction Induced by treatment with rotenone and oligomycin	Increases insulin sensitivity, glucose tolerance, and energy expenditure, and reduces body weight in mice Serum levels increase in patients with metabolic disease, potentially due to over-compensation Serum levels are reduced after adopting routine exercise	ANGPTL6’s effect on aging remains to be studied

### FGF21 and GDF15

Under normal conditions, FGF21, a mammalian mitokine, is a peptide hormone synthesized by multiple organs to control energy homeostasis. FGF21 has a plethora of metabolic functions and acts as an autocrine, paracrine, and endocrine factor. FGF21 pathways target many organs and tissues, including the liver, brown and white adipose tissues, muscles, the pancreas, and the heart. FGF21 increases energy expenditure in times of cellular stress through simultaneously acting on these tissues. In previous studies, FGF21 was shown to regulate glucose metabolism in white adipose tissue. In addition, it has been shown to act as an anti-inflammatory factor in cardiac muscle and in pancreatitis and other metabolism-related diseases. Through the regulation of aging-related genes, it is able to aid in delaying cellular aging [42]. Additionally, FGF21 is induced in instances of muscle stress and mitochondrial myopathies [43]. To this extent, increased levels of FGF21 indicate the presence of mitochondrial dysfunction, making it a prominent biomarker of human mitochondrial disorders.

Similarly, the mammalian mitokine GDF15 has been studied extensively for: (i) its expression regulated by the ISR, (ii) its metabolic regulation through suppression of food intake and controlling energy intake and (iii) its ability to prevent diet-induced hepatic steatosis [44]. Under mitochondrial stress, the eIF2α-ATF4/CHOP pathway promotes the transcription and secretion of GDF15 [45]. GDF15 also regulates metabolism. GDF15 levels increase in the liver and white adipose tissues in mouse models of diet-induced obesity (DIO). In humans, it was discovered that aerobic exercise increases the circulating levels of GDF15, contributing to fat loss. Additionally, GDF15 is secreted in adipose tissue macrophages of mice to combat the inflammatory effects of obesity-associated lysosomal stress, and prevents insulin resistance. The biological mechanism of GDF15’s metabolic actions and its targeted tissues remain unclear, and the reason why there is a high level of circulating GDF15 in obese mice has yet to be investigated [46].

FGF21 and GDF15 work synergistically to respond to hepatic mitochondrial dysfunction. Tissues in the liver are responsible for controlling hepatic metabolism, as well as regulating metabolism in other distal tissues. The mouse model of liver-specific mitochondrial stress, induced by a loss-of-function mutation of the mitochondrial ribosomal gene *CR6-interacting factor 1 (Crif1)* specifically in the liver, has significant metabolic changes such as insulin sensitivity, energy expenditure, and DIO [44]. To combat this stress and to limit the consequences of systemic metabolism modulation, hepatic adaptations of the mammalian mitokines FGF21 and GDF15 allow for local and distal energy metabolism modulation. While GDF15 regulates body fat mass and reverses hepatic steatosis, FGF21 increases insulin sensitivity and thermogenesis due to hepatic mitochondrial dysfunction. The induction of FGF21 and GDF15 together demonstrates how localized mitochondrial dysfunction non-autonomously signals distal tissues to reduce energy expenditure and equip cells to combat cellular stress. The secondary effects of this induction include systemic metabolic redefinition, phenotypically presented as a reduction in body fat mass, controlled insulin and glucose regulation and uptake, and adaptive thermogenesis [44]. This study also revealed that pyruvate dehydrogenase 4, alkaline ceramidase 2, and the switch from glucose catabolism to fatty acid utilization all increased in the liver, white adipose tissues, and gastrocnemius muscle. At the transcriptional level, FGF21 and GDF15 had the highest levels of increased expression with a 15.5- and 7.2-fold change, respectively. Beta-klotho, a co-receptor for FGF21, also demonstrated increased levels likely due to its strong association with FGF21 induction. Additionally, while gene sets for glutamine, glycogen, and fatty acid metabolism increased, those involved in steroid hormone biosynthesis and lipid metabolism decreased [44].

Of the MDPs, mitochondrial open reading frame of the 12S rRNA-c (MOTS-c), humanin (HN), and small humanin-like peptides (SHLPs) SHLP2 and SHLP3 have been identified to play a role in mitochondria stress resistance and are receiving growing attention.

### MOTS-c

MOTS-c, a 16 amino acid peptide encoded by the mitochondrial 12S rRNA gene, promotes exercise-like adaptations and has therefore been speculated to be an “exercise mitokine” [47]. In humans, higher MOTS-c levels are induced by exercise. Reynolds *et al*. found that the endogenous expression of MOTS-c increased by 1.6-fold during exercise and returned to the baseline level 4 h after exercise [48].

The location of MOTS-c is affected by stress. The serum level of MOTS-c in mice has been shown to decrease after cold stress [49]. Under resting conditions, MOTS-c in HEK293 cells is mainly found outside the nucleus. But under metabolic stress, such as glucose restriction, serum deprivation, and oxidative stress, MOTS-c translocates to the nucleus within 30 min following stress, and returns to the cytosol in 24 h. It is expressed in skeletal muscle and adipose tissues, but is also present at circulating levels in mice and in humans [49].

MOTS-c affects fat accumulation, lipogenesis, metabolism, inflammatory responses, and metabolic stress adaptation. By blocking *de novo* purine biosynthesis, MOTS-c activates AMPK, a regulator that improves energy metabolism and prevents DIO and insulin resistance. In high-fat diet-fed mice, MOTS-c reduces visceral fat accumulation and hepatic steatosis [50]. MOTS-c inhibits the expression of genes related to lipogenesis and increases glucose uptake, glucose utilization, fatty acid oxidation, and oxidative respiration. MOTS-c suppresses inflammation by inhibiting the nuclear factor-kappaB (NF-κB) and signal transducer and activator of transcription 1 (STAT1) pathway, acts to reduce the release of adhesion molecules and pro-inflammatory cytokines, such as interleukin (IL)-6, IL-1β, and tumor necrosis factor-alpha (TNFα), and increases the level of the anti-inflammatory cytokine IL-10, protecting against coronary endothelial dysfunction [51]. Through inhibiting the NF-κB pathway, MOTS-c could also activate the nuclear factor erythroid 2-related factor 2 (Nrf2)/antioxidative response element (ARE) pathway to protect against H_2_O_2_-induced inflammation and oxidative stress in H9c2 cells [52]. In addition, under metabolic stress, MOTS-c translocates to the nucleus, binds to nucleic DNA, and interacts with ARE-regulating factors [47]. Recent data in mice has shown that MOTS-c can significantly enhance physical performance in mice from 2 to 22 months of age by regulating the expression of nuclear genes related to metabolism and proteostasis, and optimize myoblast adaptation to metabolic stress [48].

MOTS-c has been postulated to affect longevity and protect against age-related diseases. The circulating levels of MOTS-c decrease with age, and MOTS-c polymorphism has been postulated to associate with human longevity. Specifically, the m.1382A>C polymorphism located in the MOTS-c-encoding mtDNA involves a K14Q mutation of MOTS-c. It is specific to the Northeast Asian population and has been proposed as a biological mechanism that explains the exceptional longevity of some Japanese people [53, 54]. When high-fat diet-fed male mice are injected with MOTS-c, they show improved glucose tolerance and reduced body weight. Interestingly, female mice injected with K14Q-MOTS-c were not affected. MOTS-c also increases the phenotype of senescence-associated secretory phenotypes, which makes senescent cells more easily detected and removed by the immune system to protect against the progression of age-related diseases [47].

HN, a peptide encoded by a small ORF in the 16S rRNA of mtDNA, has been shown to have anti-apoptosis, cyto-protective, anti-oxidation, anti-inflammatory, and neuroprotective effects [55, 56]. It also protects against inflammation-related diseases, such as Alzheimer’s disease, type 2 diabetes, cardiovascular disease, and atherosclerosis [39, 57–60]. When translated in the mitochondria, HN is composed of 21 amino acids [55]. When translated in the cytoplasm, HN is composed of 24 amino acids. Similar to MOTS-c, HN decreases the production of pro-inflammatory cytokines, such as IL-6, IL-1β, and TNFα [61]. HN has also been shown to reduce inflammation, apoptosis, and macrophage infiltration in apolipoprotein E (ApoE)-deficient mice during early stages of kidney disease [62]. HN exerts its anti-inflammatory effect partially through reducing the production of damage-associated molecular patterns [63], which are pro-inflammatory molecules that promote apoptosis. In addition, HN retains BCL-2-associated X protein (BAX) in the cytoplasm, preventing it from translocating to the mitochondria to induce apoptosis. This also has anti-inflammatory effects, as BAX activation leads to anti-inflammatory responses, such as via mitochondrial antiviral-signaling proteins, the cyclic GMP-AMP synthase-stimulator of interferon genes (cGAS-STING), inflammasomes, and NF-κB [64].

Multiple correlations between HN and age-related diseases have been identified. HN serum levels are lower in patients with Alzheimer’s disease, while the serum levels remain the same in patients with type 2 diabetes, suggesting the disease-specific modulation of mammalian mitokines [37]. Conflicting literature details the changes in circulating levels of HN that accompanies aging. Notably, an age-related decrease in HN [40] is speculated to lead to inflammation, whereas in other cases an increase of HN with age [37, 38] is speculated to counteract the detrimental effects of inflammation. It has been hypothesized that the adaptive mammalian mitokine response observed in healthy aging is lost in age-related diseases, and that GDF15, FGF21, and HN might act together only during physiological aging in the absence of age-related diseases [37]. Interestingly, circulating HN levels in children of centenarians are also higher than age-matched controls, suggesting potential genetic polymorphisms that control HN expression and subsequently human aging [65].

### SHLPs

SHLPs are mitochondria-derived peptides encoded by small open reading frames in the mitochondria-encoded 16S rRNA. To date, six SHLPs have been identified, of which SHLP2 and SHLP3 have been shown to exhibit similar properties as HN. Specifically, they reduce apoptosis, decrease generation of ROS, improve mitochondrial metabolism, increase glucose uptake, suppress hepatic glucose production, and increase cell viability [66]. In addition, they enhance 3T3-L1 pre-adipocyte differentiation [55]. SHLP2 and SHLP3 are mainly expressed in the liver, kidney, and spleen. SHLP2 has been found to increase the number of pancreatic cells [67]. SHLP2 is also detected in the muscles, and SHLP3 is also detected in the brain. Extensive research is still required to understand the exact mechanism by which SHLPs act. SHLP2 has been suggested to regulate leptin levels, while SHLP3 regulates IL-6 and monocyte chemotactic protein-1 (MCP-1), in addition to leptin levels [55]. Interestingly, SHLP2 has been shown to protect against amyloid beta 1-42 (Aβ1-42)-induced cell death and counteract Alzheimer’s disease. The circulating level of SHLP2 has been shown to decrease with age [67].

### ADM2

ADM2 is an endogenous bioactive peptide induced in response to mitochondria stress [68]. Its transcription is also regulated by the ISR transcription factor ATF4. ADM2 affects the beiging of white adipose tissues and insulin resistance, and it has been found to correlate with age and memory retention in mice. Overexpression of ADM2 in adipocytes induces upregulation of uncoupling protein 1 (UCP1) and beiging of white adipose tissue [69]. Treatment with recombinant ADM2 inhibits obesity-induced insulin resistance in mice through deactivating adipose CD4^+^ T cells [70]. In patients with obesity, ADM2 signaling is found to be suppressed in adipose tissues and is associated with lower receptor expression and ligand availability. This has been postulated to contribute to insulin resistance [71]. In addition, ADM levels are elevated in aging human brains, and ADM2 knockout mice showed better memory retention than control mice [41]. Yet, ADM levels have been found to decrease with age in mice [72].

### ANGPTL6

ANGPTL6 has various metabolic benefits. It increases insulin sensitivity, glucose tolerance, and energy expenditure, and reduces body weight in mice [73]. In cultured adipocytes, ANGPTL6 is induced by OXPHOS dysfunction as a result of *Crif1* knockout, or treatment with oligomycin, which inhibits the ATP synthase, and rotenone, which inhibits complex I of the mitochondrial respiratory chain [74]. ANGPTL6 is also required for Fgf21 expression in adipocytes. Paradoxically, an increased level of ANGPTL6 has been found in patients with metabolic diseases, potentially due to over-compensation. Similarly, both high-fat diet feeding and leptin treatment are associated with higher levels of ANGPTL6 expression, while ANGPTL6 levels are decreased by exercise [75]. A recent randomized control trial showed that patients with metabolic syndrome have higher levels of ANGPTL6, and the level is reduced after a 12-week exercise regimen. This suggests that exercise could improve metabolism and thus reduce the need for compensatory upregulation of mammalian mitokines [76].

## Bacterial quorum sensing as an origin of intercellular mitochondrial communication?

**Figure 2:**
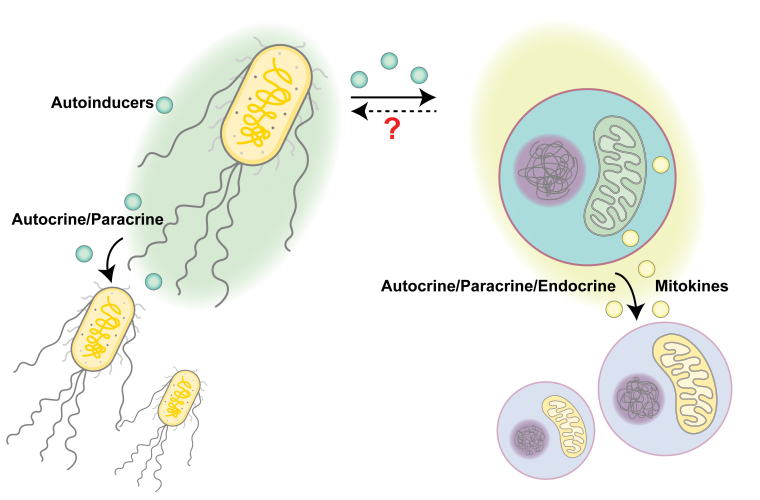
Comparison between bacterial QS and intercellular mitochondrial communication. Bacteria may secret autoinducers to coordinate other individuals nearby to achieve synchronized functions at the population level. In comparison, mitochondrial stress signaling may be transmitted via mitokines to elicit mitochondrial stress responses or metabolic remodeling in recipient cells that are either proximal or distal. Bacterial autoinducers may cause mitochondrial stress, yet it is unclear whether mitokines may modulate bacterial behaviors.

Considering that the origin of mitochondria is aerobic prokaryotes, is it possible that the widely observed inter-tissue communication of mitochondrial stress could be descendants of ancient bacterial communications? In this section, we will compare bacterial communications with mitochondrial communications (Fig. 2), hoping to initiate a new perspective on understanding mitokine-mediated intercellular mitochondrial communications.

Although historically thought of as single living organisms, bacteria can produce and sense communication signals within the same species or with other bacterial species. This process is termed quorum sensing (QS) and the secreted molecules, usually oligopeptides or metabolites, are called autoinducers. Like the UPR^MT^ that induces gene transcriptional changes to cope with mitochondrial stress, QS is a regulatory scheme that controls gene transcription in response to cell-population density changes. When the population density increases, autoinducers accumulate and reach a certain threshold to initiate specific gene expression programs. These programs may help coordinate behaviors within the population, including the onset of bioluminescence, production of virulence factors, and formation or collapse of biofilms [77]. A common feature of QS-induced responses is the synchronized behaviors: autoinducers often promote the expression of autoinducer synthases to drive the whole community into the QS mode via a positive feedback loop. The advantage for such coordinated behaviors is to accomplish functions that cannot be achieved by an individual cell alone, such as generating sufficient amounts of nutrient-scavenging molecules, toxins, and immune suppressants to alter the environment for better survival and expansion.

Similarly, inter-cellular communication of mitochondrial stress also has a synchronizing role in coordinating or amplifying stress responses throughout the whole body. As discussed earlier, inducing mitochondrial stress in neurons or the germline is sufficient to elicit a whole-body mitochondrial stress response in worms. This coordinated stress response across tissues may prepare the whole organism for potential mitochondrial toxins or environmental stress that is sensed by “whistler” cells. Like bacterial QS, a whole-body level response can be more efficient in maintaining the homeostasis of the organism than restricting the stress responses within those already affected cells.

Intriguingly, some mitokines directly originate from mitochondria to achieve inter-cellular communication among the mitochondrial community, supporting the idea that mitochondria from different cells may actively communicate with each other. As discussed above, mitochondrial genome encodes small peptides, such as MOTS-c, HN, and SHLPs. Mitochondrial stress induced by exercise, glucose restriction, or tert-butyl hydrogen peroxide treatment can activate MOTS-c and cause its nuclear translocation from the mitochondria, as well as its release into the circulation [47, 48]. Exogenous MOTS-c treatment via intraperitoneal injection can improve the physical capacity and the healthspan of old mice [48], suggesting that increased MOTS-c levels in circulation may signal to improve tissue functions in a non-autonomous manner. As another example, mitochondrial stress-induced HN exerts its effect both cell-autonomously via binding to intracellular receptors such as BAX to inhibit apoptosis, and cell-non-autonomously via being released and binding to cell-surface receptors or relocating to mitochondria of recipient cells [78]. Due to their healthspan-extending effects, HN and its mimetics have been developed in the past decade for treating degenerative diseases, including neurodegeneration, cardiovascular diseases, and diabetes [78]. The functions of these mitochondria-derived signaling peptides are complicated, potentially due to long-term co-evolution of the mitochondria and the host organism. It remains an exciting field to study how these peptides regulate mitochondrial homeostasis and other cellular functions under metabolic stress conditions, and how they may signal between cells to coordinate the stress responses.

It is interesting to note that bacterial autoinducers may directly impact mitochondria, although it is unclear whether such interactions are an acquired feature during host-microbiome co-evolution or have already existed since endosymbiosis occurred. For example, colanic acid is a polysaccharide secreted by many enterobacterial species, including *E. coli*. Under stress conditions, colanic acid forms a capsule to protect bacteria. Using a single-gene knockout *E. coli* mutant library, Han *et al.* tested how the lifespan of *C. elegans* might be affected by the quality of their food and microbiota, i.e. various *E. coli* mutant strains in this experiment [79]. They found that mutations causing increased production of colanic acids prolonged the lifespan of worms [79]. Mechanistically, purified colanic acid is sufficient to induce mitochondrial fragmentation in worm intestine, and extends animal lifespan via enhancing the UPR^MT^ signaling [79]. It seems that this extracellular matrix molecule may directly function as a signaling molecule to modulate host mitochondrial homeostasis, although the precise mechanism, such as the cognate receptor, in host cells is still unclear. Another example is the autoinducer produced by *Pseudomonas aeruginosa* called N-(3-oxo-dodecanoyl)-l-homoserine lactone (3OC12). Concentrations of over 600 μmol/L 3OC12 have been detected in *P. aeruginosa* biofilms *in vitro* [80]. Due to its lipophilicity, 3OC12 can freely pass through lipid membranes via passive diffusion. Mammalian cells express lactone hydrolyzing enzymes called paraoxonases (PONs) that may convert 3OC12 from its original lactone form into its carboxylic acid counterparts. In contrast to the lactone form of 3OC12, the 3OC12 acids cannot freely pass through membrane structures. The paraoxonase PON2 predominantly localizes to the ER and the inner mitochondrial membrane [81, 82]. Therefore, mitochondrial PON2 can cause formation and accumulation of 3OC12 acids within mitochondria, which in turn causes mitochondrial acidification, decline of mitochondrial membrane potential, release of calcium and cytochrome *c,* and apoptosis [83, 84].

Conversely, mitokines may also regulate bacterial behaviors in an indirect manner via regulating host immune responses. For example, the mitochondria-derived peptide MOTS-c promotes the phagocytic and bactericidal abilities of macrophages, which helps decrease bacterial loads and improve the survival rate of *Staphylococcus aureus*-challenged mice [85]. However, whether mitokines may directly affect bacteria is questionable. Generally, mitokines are hormone-like signaling molecules that are produced in a very low amount to implement their sensitive signaling functions. In contrast, autoinducers have a higher signal threshold. They require accumulating doses as an indicator of high cell density. Therefore, it seems unlikely that mitokines may be directly sensed by bacteria and function as host-derived autoinducers.

Besides the signal threshold, another major difference between autoinducer signaling and mitokine signaling is the signal calling distance. Bacterial communication is primarily an intra-aggregate communication system and heavily relies on the population density. Using micro-3D printing and scanning electrochemical microscopy techniques, Connell and colleagues reported that QS-dependent behaviors may occur in aggregates with as few as 500 cells. Long-range inter-aggregates communication requires more cells and higher concentration for diffusion. Larger than 2000 cells are required to stimulate QS in a neighboring aggregate that is 8 μm away [86]. In contrast, mitochondrial inter-cellular or inter-tissue communication has a much longer calling distance. Mitokines in higher organisms may make use of the far-reaching nervous system or the circulatory system to regulate mitochondrial homeostasis in distal tissues. This process seems to rely less on the density of stressed mitochondria or the concentration of mitokines.

In summary, there is a lack of strong evidence to speculate that the mitokine signaling machinery directly originates from bacterial QS based on existing findings. However, knowledge in QS may illuminate research of mitochondrial inter-cellular communications. Just as quantitative analyses performed in bacterial QS research, different types and intensity of mitochondrial stress may lead to various mitokine responses regarding the types of mitokines and recipient tissues involved, as well as the responses of the recipient tissues. Moreover, whether autoinducers may mimic mitokines to modulate host metabolism is also an intriguing question to investigate.

## Outlook

Mitochondrial homeostasis is closely related to organismal metabolic functions. It is worth noting that mitochondrial stress may not only affect the focal tissues, but also coordinate with the rest of the body through long-range communications. This knowledge may promote discoveries of mitokine-based biomarkers for assessing organismal metabolic conditions, as well as the development of novel preventative and therapeutic strategies for improving metabolic health.
